# Early evaluation of corneal collagen crosslinking in *ex-vivo* human corneas using two-photon imaging

**DOI:** 10.1038/s41598-019-46572-3

**Published:** 2019-07-15

**Authors:** Ana Batista, Hans Georg Breunig, Tobias Hager, Berthold Seitz, Karsten König

**Affiliations:** 1 Saarland University, Department of Biophotonics and Laser Technology, Campus A5.1, 66123 Saarbruecken, Germany; 2 JenLab GmbH, Johann-Hittorf-Straße 8, 12489 Berlin, Germany; 3 Saarland University, Department of Ophthalmology, Medical Center, 66421 Homburg, Saar Germany; 4 Lions Cornea Bank Saar-Lor-Lux, Trier/Westpfalz, Medical Center, 66421 Homburg, Saar Germany

**Keywords:** Medical imaging, Biomedical engineering, Corneal diseases, Nonlinear optics, Biophotonics

## Abstract

The clinical outcome of corneal collagen crosslinking (CXL) is typically evaluated several weeks after treatment. An earlier assessment of its outcome could lead to an optimization of the treatment, including an immediate re-intervention in case of failure, thereby, avoiding additional discomfort and pain to the patient. In this study, we propose two-photon imaging (TPI) as an earlier evaluation method. CXL was performed in human corneas by application of riboflavin followed by UVA irradiation. Autofluorescence (AF) intensity and lifetime images were acquired using a commercial clinically certified multiphoton tomograph prior to CXL and after 2*h*, 24*h*, 72*h*, and 144*h* storage in culture medium. The first monitoring point was determined as the minimum time required for riboflavin clearance from the cornea. As control, untreated samples and samples treated only with riboflavin (without UVA irradiation) were monitored at the same time points. Significant increases in the stroma AF intensity and lifetime were observed as soon as 2*h* after treatment. A depth-dependent TPI analysis showed higher AF lifetimes anteriorly corresponding to areas were CXL was most effective. No alterations were observed in the control groups. Using TPI, the outcome of CXL can be assessed non-invasively and label-free much sooner than with conventional clinical devices.

## Introduction

Corneal collagen crosslinking (CXL) is a medical procedure used in clinical practice to strengthen the mechanical stability of the cornea^1,2^. The process is based on the photodynamic interaction between a photosensitizer, typically riboflavin, and ultraviolet A (UVA) light which creates reactive oxygen species (ROS). These induce the formation of new crosslink bounds between collagen molecules and fibrils, thereby strengthening the tissue^1,2^. In clinical practice, CXL is commonly applied to patients diagnosed with keratoconus and ectasia secondary to laser refractive surgery. Recently, its feasibility to treat myopia, corneal edema, and infectious keratitis has also been demonstrated^1–4^.

Although the safety and reliability of CXL for halting the progression of keratoconus has been demonstrated in several clinical trials^5–10^, in the past years, reports of treatment failure have been presented^11–13^. The treatment outcome can be evaluated by assessing the patient’s corneal topography as well as the uncorrected and best corrected visual acuities^1^ over a period of several months. The first examination is performed not earlier than a one month after CXL. The outcome of CXL can also be assessed, several weeks after treatment, based on *in vivo* confocal microscopy (IVCM) by evaluating the corneal stroma reflectivity, corneal edema, and keratocyte apoptosis^1,14–16^, or based on anterior-segment optical coherence tomography (AS-OCT) through the detection of a hyperreflective line, *i.e*., the “corneal demarcation line”, which marks the transition between CXL and non-CXL areas^17–19^.

CXL failure is considered when the progression of disease cannot be halted. The reported rates of treatment failure go up to 16.5%^11,12^. Although, the course of treatment in case of failure is not yet defined, a second CXL procedure is one of the options^13^. This requires again corneal de-epithelialization which leads to patient’s pain, burning sensation, and tearing discomfort for several days^1^. An earlier evaluation of the outcome of CXL could lead to a faster intervention, such as immediate CXL redo, thereby, avoiding additional pain for the patient.

In this study, we propose two-photon imaging (TPI) to early evaluate the outcome of CXL. In our previous publications we have demonstrated that TPI can provide valuable information on the cornea^20–22^, inaccessible with current clinical devices, that can improve the evaluation of corneal donor buttons prior to transplantation^23^ or corneal disease diagnosis^24^. In this study, we show that based on TPI, CXL-induced changes to the corneal stroma can be assessed non-invasively, label-free, shortly after treatment, and with fast acquisition times.

## Material and Methods

### Human corneas

Human corneal donor buttons, unsuitable for transplantation, were obtained from the Lions Cornea Bank Saar-Lor-Lux, Trier/Westpfalz at the Department of Ophthalmology, Saarland University Medical Center, Homburg/Saar, Germany. A total of 39 donor buttons with average storage times of (17 ± 14) weeks were used. From stroma to endothelium, the samples were on average 962 *μm* thick, with thicknesses varying between approximately 675 and 1190 *μm*. No significant changes were observed during the experiment duration (thickness changes below 10% in 144 *h*). Thickness data were obtained using multiphoton tomography. Prior to the experiments, samples were stored in culture Medium II with Dextran T500 (#F9017, Biochrom GmbH, Berlin, Germany) supplemented with 5% new-born calf serum (#S0415, Biochrom GmbH, Berlin, Germany) at 34 °C in a 5% CO_2_ atmosphere.

This study was approved by the ethics committee of the University of Saarland and it was conducted according to the principles for research use of human tissue of the World Medical Association Declaration of Helsinki. Written and informed consent for the use of their tissue for scientific research was obtained for all subjects.

### Accelerated corneal collagen crosslinking

**Figure 1:**
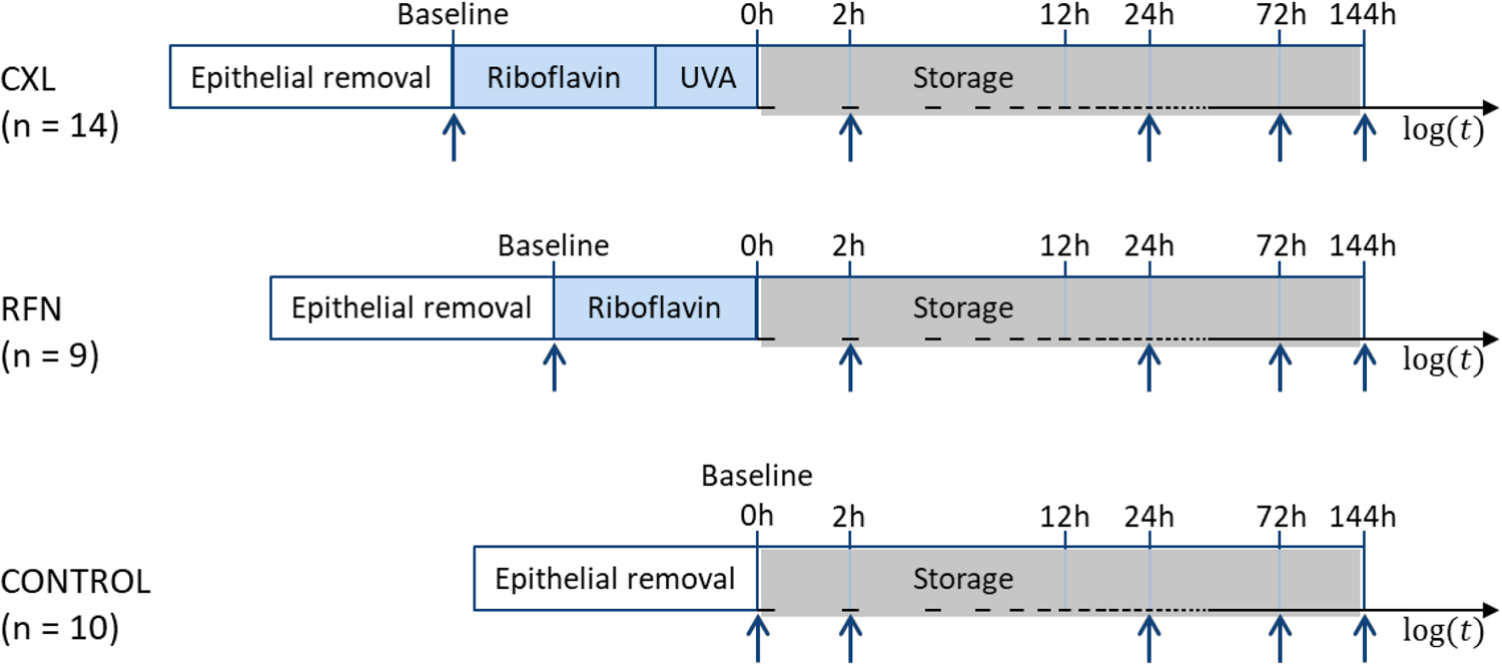
Schematic representation of the timing of crosslinking experiments. The vertical arrows indicate time points of two-photon imaging. CXL – corneal collagen crosslinking, RFN – riboflavin.

The samples were divided into 3 groups and treated as illustrated in Fig. 1:

- *CXL (n* = *14):* CXL was performed, after corneal de-epithelialization using a blunt hockey knife, through the application of 0.1% riboflavin solution (Vibex rapid™ Avedro, Inc., MA, USA) every 2 *min* for 20 *min* followed by irradiation with an UVA light source for 10 *min* using an in-house adapted CXL system consisting of a 365 *nm* light-emitting diode (LED) mounted on an inverted microscope. A total energy dose of 7.2 *J*/*cm*^2^ was applied (irradiance of 12 *mW*/*cm*^2^). The feasibility of our setup to perform CXL was confirmed by comparing with samples treated using a commercial CXL system. Both systems yielded similar results, which showed the capability of our setup to induce CXL^25^.
- *RFN (n* = *9):* de-epithelialized corneal donor buttons were infused with 0.1% riboflavin solution (every 2 *min* for a total of 20 *min*) without UVA irradiation.
- *Control (n* = *10):* the epithelial layer was removed using a blunt hockey knife without further treatment.

TPI acquisition was performed for all groups immediately after epithelial removal (baseline) and after 2 *h*, 24 *h*, 72 *h*, and 144 *h* storage. Riboflavin clearance from the tissue was achieved by 2 *h* immersion in culture Medium II with Dextran T500 supplemented with 5% new-born calf serum. Between measurements, samples were stored as described above. For image acquisition, samples were mounted on an artificial anterior chamber. Live cell imaging solution (#A14291DJ, Life Technologies, USA) was used to hydrate them.

### Multiphoton tomography

**Figure 2:**
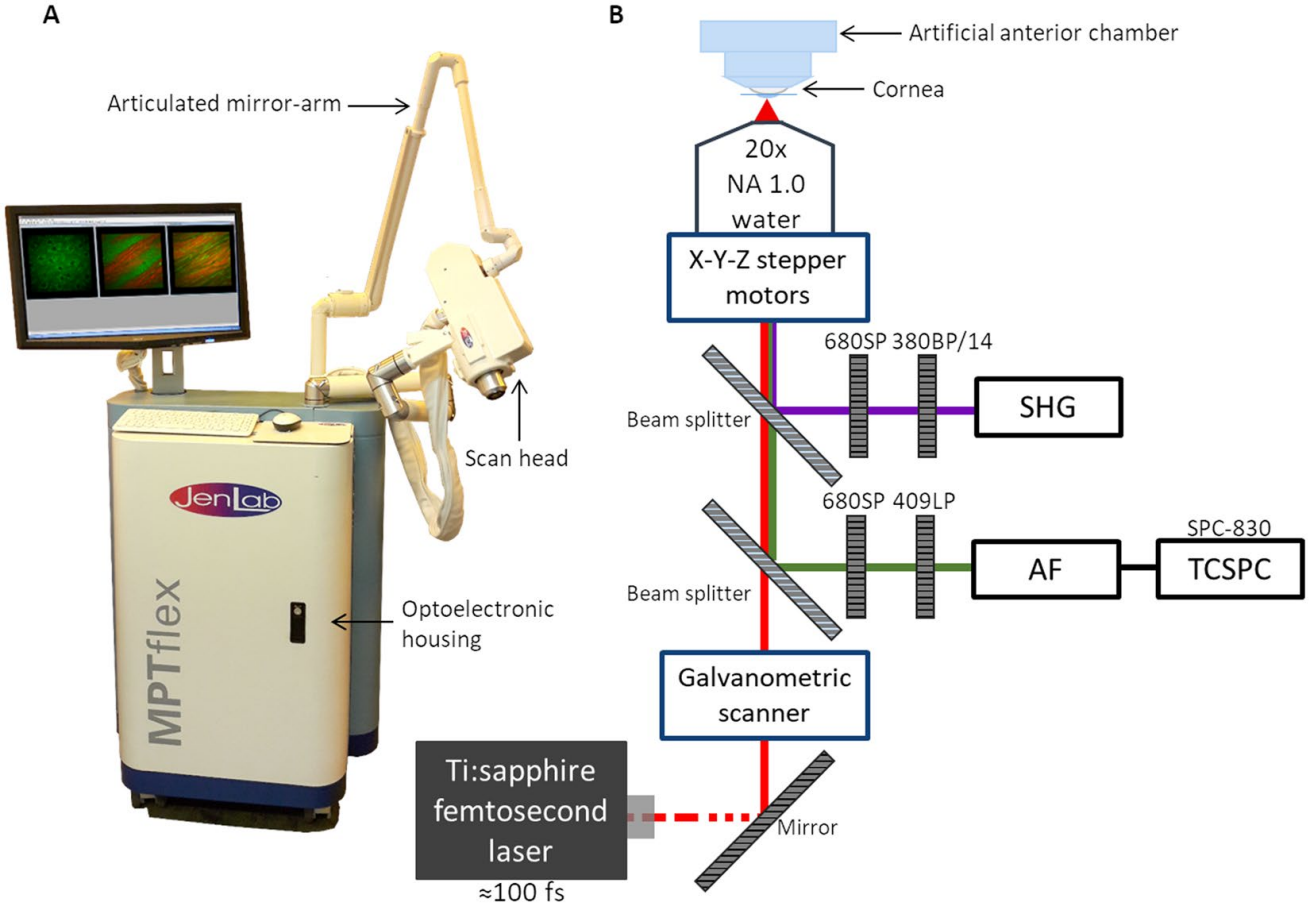
Multiphoton tomograph MPT*flex* (JenLab GmbH, Berlin, Germany) used for image acquisition (**A**) and schematic representation of the instrumental optical setup (**B**).

Image acquisition was performed using the commercial tomograph MPT*flex* (JenLab GmbH, Berlin, Germany; Fig. 2) clinically certified for human skin imaging. The system consists of a movable housing that includes the laser and other optoelectronic components (optoelectronic housing), a flexible 360° measurement head that enables TPI measurement in multiple angles, and an articulated mirror-arm that guides the laser light of a 80 MHz tunable near infrared (NIR) Ti:sapphire laser with pulse widths of 100 femtosecond (*fs*) (Fig. 2). The laser beam is focused onto the sample by a 20x NA 1.0 water immersion objective with long working distance (1700 *μm*) which allows to image the entire corneal thickness. The measurement head contains galvanometric *x-y* scanners and stepper motors to change the imaged regions in *x*, *y*, and *z* directions, respectively. Signals are collected in reflection geometry by the objective and detected in two spectral channels by photomultiplier tubes (PMT) also placed inside the measurement head. With this configuration second-harmonic generation (SHG) and autofluorescence (AF) signals are detected simultaneously (Fig. 2B). The PMT in the AF channel is coupled to a time-correlated single photon counting (TCSPC) module. Image acquisition of the cornea samples was performed using a fixed excitation wavelength of 760 *nm*.

At each time point, six *x-z* cross-sectional images and *en-face* images of two non-overlapping regions were acquired. For the latter, images up to a depth of 300 *μm*, with 5 *μm z*-steps in-between were recorded. Image acquisition times were typically 6.2 *s* for each *en-face* image with 512 × 512 *pixels*, and 68 *s* for each cross-sectional image with 512 × 1024 *pixels* (field-of-view = 600 × 1200 *μm*^2^). The laser power was varied between 20 and 50 *mW* as a function of imaging depth. Images were acquired with a lateral and axial resolutions of 450 *nm* and of 2–3 *μm*, respectively.

### Image analysis

The influence of the CXL on the corneal stroma was evaluated using the stroma AF intensity and lifetime. Variations in the stroma AF intensity (Δ*I*) were computed as:

1 $${\rm{\Delta }}I={I}_{t}-{I}_{i},$$

where *I*_*i*_ and *I*_*t*_ are the initial stroma AF intensity (baseline) and the AF intensity at a time *t*, respectively. CXL-induced changes to Δ*I* were measured at *t* = 2, 24, 72, 144 *h* after treatment. At each time point, the AF intensity was obtained from to total number of photons collected. To determine Δ*I* during riboflavin clearance from the tissue, the signal intensity was measured at *t* = 0, 30, 60, 90, 120 *min* after riboflavin application.

The corneal stroma mean AF lifetime was computed using the commercial software SPCImage (Becker & Hickl GmbH, Berlin, Germany). Briefly, to retrieve the AF lifetime, the obtained TCSPC histogram was fitted, after deconvolution with the instruments response function (IRF), by an exponential function in the form:

2 $$F(t)=\sum _{i=1}^{n}{a}_{i}{e}^{-t/{\tau }_{i}},n=2$$

where *F*(*t*) is the fluorescence intensity at time *t*, and *a*_*i*_ is the relative contribution of the fluorescence lifetime *τ*_*i*_. The mean AF lifetime (*τ*_*m*_) was computed as the average of all lifetime components (*τ*_*i*_) weighted by their relative contributions (*a*_*i*_). The IRF was deduced from the measured SHG signal profile generated by crystallized urea.

### Statistical analysis

The statistically significance of alterations on the stroma AF intensity variation (Δ*I*) and lifetime due to CXL were determined using the commercial software GraphPad Prism version 6.05 (GraphPad Software Inc., California, USA). The results are shown as averages and standard deviations (SD). The parametric *t-test* was used. In the cases where data did not follow a Gaussian distribution, the non-parametric equivalent, *Mann-Whitney U test*, was used instead. We considered *p* values lower than 0.05 as being significant.

## Results

### Two-photon excited autofluorescence and riboflavin fluorescence

In the corneal stroma, endogenous sources of fluorescence include collagen reduced coenzymes nicotinamide adenine dinucleotide and nicotinamide adenine dinucleotide phosphate (NAD(P)H) and negligible amounts of flavin mononucleotide and flavin adenine dinucleotide (flavins). These fluorophores have fluorescence emission maxima around 485 *nm*, 460 *nm*, and 525 *nm* respectively^26–28^. Riboflavin’s fluorescence, with a maximum emission around 530 *nm*, spectrally overlaps with that of endogenous fluorophores of the cornea. Thus, changes of the AF intensity and lifetime were analyzed after riboflavin clearance from the tissue.

**Figure 3:**
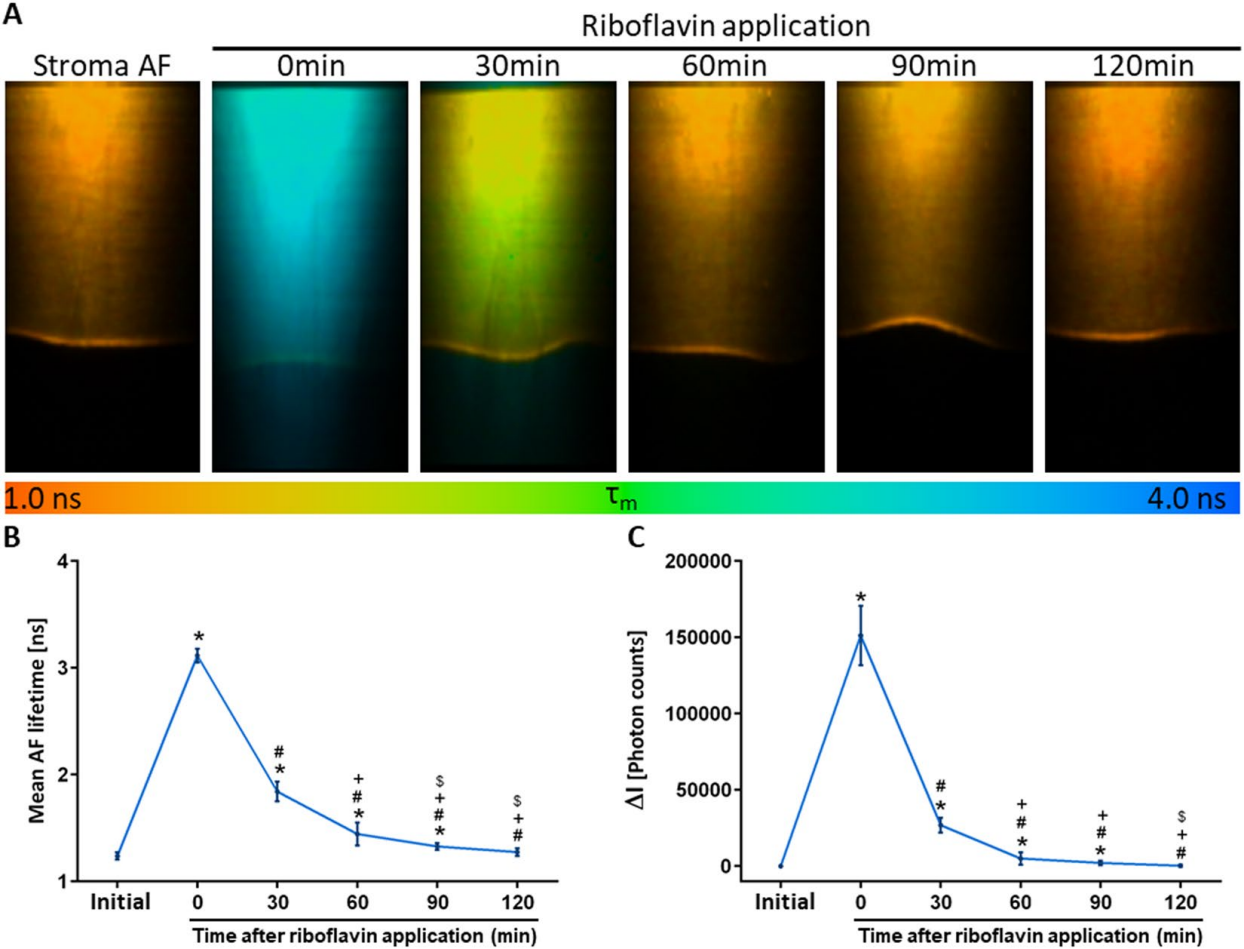
Cross-sectional autofluorescence (AF) lifetime images of the cornea during riboflavin clearance (**A**). Average changes of the mean AF lifetime (**B**) and of the AF intensity variation (Δ*I*) over time (**C**). Cross-sections cover an area of 600×1200 *μm*^2^. Statistical significance was computed using *Mann Whitney U test*. *statistically significant at ρ < 0.05 compared with baseline; ^#^statistically significant at ρ < 0.05 compared with *t* = 0 *min*; ^+^statistically significant at ρ < 0.05 compared with *t* = 30 *min*; ^$^statistically significant at ρ < 0.05 compared with *t* = 60 *min*.

The minimum time required for riboflavin to completely clear out from the tissue was determined in 4 corneal donor buttons using TPI. The AF intensities and lifetimes were monitored prior to riboflavin application and every 30 *min* for a period of 2 *h*. The observed changes in Δ*I* and AF lifetime are shown in Fig. 3.

Prior to the application of the photosensitizer, the tissue had a bi-exponential AF decay with mean AF lifetime of (1.24 ± 0.03) *ns*. Immediately after its application (*t* = 0 *min*), significant increases in both the average AF lifetime and average Δ*I* were observed (Fig. 3). Additionally, at *t* = 0 *min*, the tissue exhibited a single exponential decay of (3.12 ± 0.06) *ns*. This is good agreement with the fluorescence lifetime recorded for the pure 0.1% riboflavin solution ((3.37 ± 0.06) *ns*). After 30 *min* of storage, riboflavin clearance from the cornea become noticeable. Two areas with distinct lifetimes were visible which indicated shorter AF lifetimes anteriorly than posteriorly (Fig. 3A). The average lifetime and Δ*I* at *t* = 30 *min* were significantly lower than those measured at *t* = 0 *min*, but still significantly higher than those observed prior to riboflavin application (Fig. 3B,C). The average AF lifetime and Δ*I* continued to considerably decrease after 60 *min *and 90 *min* storage and values equivalent to initial AF values were reached after 120 *min* storage (Fig. 3).

### CXL-induced alterations to the corneal stroma AF

**Figure 4:**
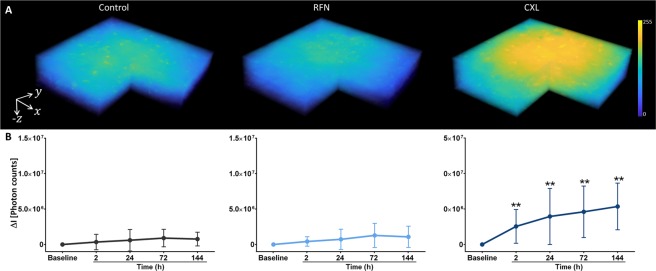
3D representations of the donor corneal samples for control, RFN and CXL groups after 72 *h* storage. Volumes cover 300 × 300 × 300 *μm*^3^ and were reconstructed from individual and sequential autofluorescence (AF) intensity images recorded with 5 *μm* intervals along the ***z*** axis. Average differences on AF intensities variation (Δ*I*) for all groups over time. Statistical significance was computed using *t-test*. **statistically significant at ρ < 0.01.

**Figure 5:**
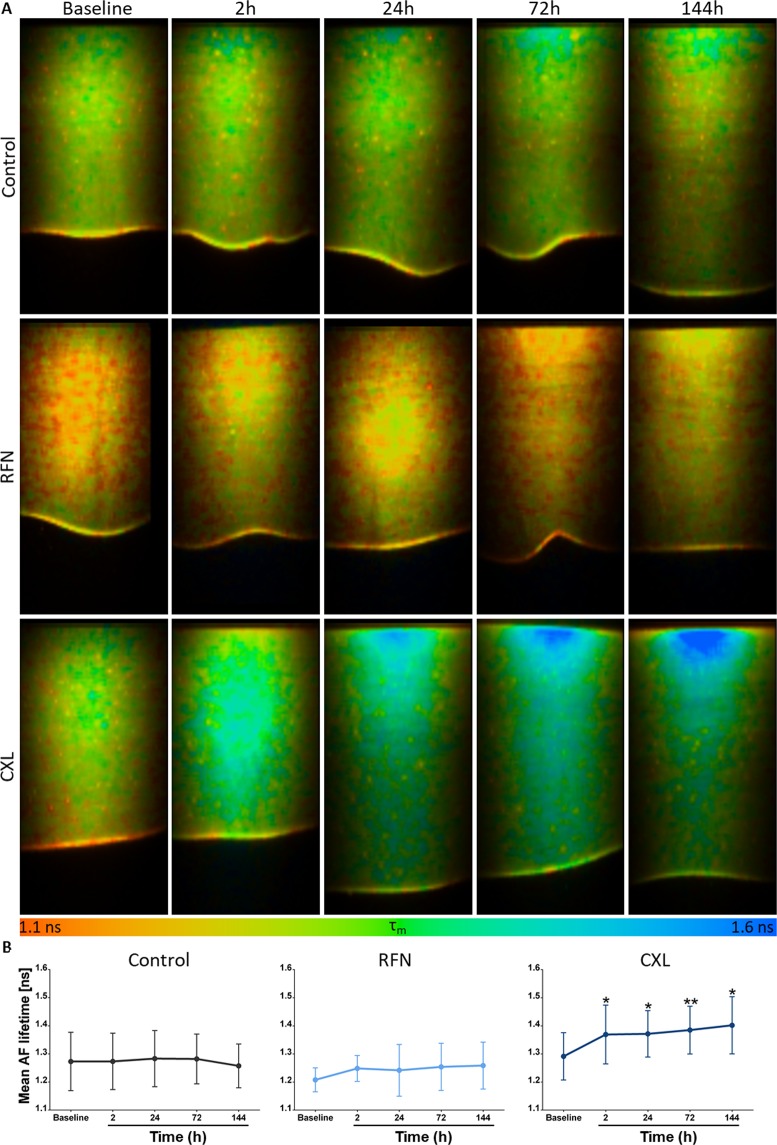
Cross-sectional autofluorescence (AF) lifetime images of the cornea at baseline, 2 *h*, 24 *h*, 72 *h*, and 144 *h* for control, RFN and CXL groups (**A**) and average variations in the mean AF lifetime over time (**B**). Cross-sections represent an area of 600 × 1200 *μm*^2^. Statistical significance was obtained using *t-test*. *statistically significant at ρ < 0.05; **statistically significant at ρ < 0.01.

The changes induced by the CXL to the corneal stroma AF intensity and AF lifetime are depicted in Figs 4 and 5, respectively. Figure 4A shows 3D representations, reconstructed from individual and sequential *en-face* AF intensity images, of corneas in CXL, RFN, and control groups after 72 *h* storage. The average changes in Δ*I* are shown in Fig. 4B. For control and RFN corneas, the AF intensity was constant over time, whereas after CXL, Δ*I* was significantly higher as soon as 2 *h* after treatment and remained significantly higher during subsequent measurements.

Representative cross-sectional AF lifetime images of corneas of control, RFN, and CXL groups, and their average mean AF lifetime over time are shown in Fig. 5. Following CXL treatment, a significant increase in the tissue mean AF lifetime was observed. This increase was observed for all measured time points. No significant changes on the average AF lifetime of consecutive measurements were observed for samples in the control and RFN groups (Fig. 5).

**Figure 6:**
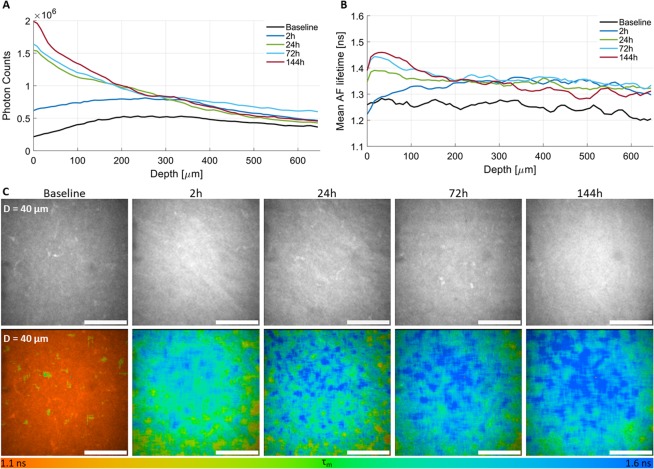
Corneal stroma autofluorescence (AF) intensity (**A**) and mean AF lifetime (**B**) after CXL as a function of depth for baseline, 2 *h*, 24 *h*, 72 *h*, and 144 *h*. AF intensity and corresponding AF lifetime images for the same corneal depth as function of time (**C**). Scale bar = 100 *μm*.

Figure 6 shows depth-dependent changes on the AF lifetime and intensity induced by CXL. Interestingly, 2 *h* after CXL, both the tissue AF lifetime and intensity increased uniformly with depth. However, with time both parameters become progressively higher in the anterior than posterior stroma (Fig. 6A,B). Additionally, for the same corneal depth the AF intensity and lifetime increase progressively with time (Fig. 6C).

## Discussion

In this study, we evaluated the outcome of CXL for corneal donor buttons using TPI. We showed that CXL induces significant increases in AF intensity and lifetime of the corneal stroma. No significant alterations were observed for control and RFN groups. Moreover, a depth analysis of the stroma AF demonstrated the feasibility of TPI to determine the transition between crosslinked and non-crosslinked areas.

So far, the clinical evaluation of the outcome of CXL is typically performed several weeks after treatment. Methods capable of assessing CXL outcome soon after treatment are important, since they could provide a faster determination of treatment efficiency which may allow immediate CXL redo in case of failure. In the recent years, efforts to obtain such an examination tool have been carried out. Namely, the feasibility of Brillouin microscopy to assess corneal alterations soon after CXL was been shown *ex vivo*^29–31^. Based on this imaging modality the Young’s modulus of the tissue can be retrieved, and a significant increase in the corneal stiffness (associated with an increase in the Brillouin shift) has been reported soon after CXL^29–32^. Nevertheless, Brillouin microscopy has a major drawback, since long acquisition times are necessary to obtain a single image (between 30 *min* and 120 *min* per image)^29–31^. The feasibility of SHG imaging to evaluate changes to the collagen fibers organization due to CXL has also been demonstrated^3,33–36^. The increase in stromal stiffness due to the creation of new crosslinks between collagen induced by CXL leads to changes in the collagen fibers organization. In 2011, Bueno *et al*. observed such changes in SHG images of porcine and bovine corneas immediately after CXL^33^. The evaluation of the collagen fibers of forward-detected SHG images showed an increase in the fiber waviness after treatment^34^. Recently, the collagen-fiber orientations of healthy, keratoconus, and keratoconus corneas after CXL have also been evaluated and compared^36,37^.

The focus of this paper is to demonstrate the potential of analyzing the stroma AF as a tool for early assessment of the outcome of CXL. So far, a quantitative time- and depth-dependent analysis of CXL-induced changes to the human stroma AF intensity and lifetime has not yet been performed, although one group reported on stroma AF lifetime alterations in rabbit eyes^38^ two-weeks after performing CXL.

The fluorescence of the photosensitizer used to perform CXL (riboflavin) must be separated in order to evaluate CXL-induced changes on the stroma AF. Riboflavin fluorescence emission overlaps with that of the tissue endogenous fluorophores, thus making a spectral separation of the fluorescent components difficult. Nevertheless, fluorophore’s separation based on their fluorescence lifetime is, in principle, possible. Whereas riboflavin has a fluorescence lifetime of (3.37 ± 0.06) *ns*, NAD(P)H has two AF lifetime components, a fast one in the picosecond range associated with its free component and typical lifetimes of 2.2 *ns* to 2.4 *ns* associated with its protein-bound component^39^. For collagen, a fast lifetime component around 0.3 *ns* and a slow component between 2.0 and 2.5 *ns* have been reported^40^. In this study, CXL-induced changes to the stroma AF were evaluated after riboflavin was absent from the tissue. Riboflavin clearance was monitored based on the fluorescence intensity and lifetime. This fluorophore leads to a strong increase in the measured fluorescence intensity and lifetime (Fig. 3). At *t* = 0 *min*, an 9-fold signal intensity increase was observed due to the riboflavin fluorescence. After 30 *min*, larger lifetimes were observed posteriorly, indicating a decreasing riboflavin concentration in the anterior stroma. Intensity and lifetime values returned to values equivalent to those of stromal intrinsic fluorescence after 120 *min* (Fig. 3) indicating the waiting time required for the riboflavin to clear out of the tissue under our experimental conditions. The time point of first CXL evaluation was set to fit this condition. For an *in vivo* application, the time required for riboflavin clearance in patients must also be quantified in the future.

The stroma AF intensities and lifetimes were evaluated prior to CXL (baseline) and 2 *h*, 24 *h*, 72 *h*, and 144 *h* after the procedure. Additionally, untreated corneas (control) and corneas treated only with riboflavin (RFN) were monitored at the same time points. For control and RFN groups no changes were observed in the stroma AF intensity and lifetime over time. However, in CXL corneas, significant increases in the AF intensity and in the AF lifetime were observed as soon as 2 *h* after treatment, which remained elevated in subsequent measurements (Figs 4 and 5). An increase in both values has also been observed in the stroma of rabbit’s corneas 2 weeks after CXL^38^. The observed increases are correlated to an increase in the number of crosslink bounds between collagen molecules. It has been found *in vitro* that collagen gels with reduced crosslinks have lower AF lifetimes, whereas models of enhanced CXL exhibit a mean AF increase of 9%^41,42^. Therefore, the observed increase in the stroma AF lifetime after CXL indicates an increase in the number of new crosslinks bound between collagen, *i.e*., a positive treatment outcome. Although alterations to keratocytes metabolic activity may also influence the stroma AF lifetime, we have observed that these cells had a small contribution to the overall stroma AF signal.

A depth-dependent analysis of the AF intensity and lifetime demonstrated the feasibility to discriminate between crosslinked and non-crosslinked areas (Fig. 6). A generalized increase in the AF intensity and AF lifetime was observed 2 *h* after CXL. After 24 *h*, 72 *h*, and 144 *h* the increase in both parameters was more pronounced for the anterior stroma. This indicates that a higher number of new crosslink bounds were induced anteriorly, corresponding to the areas were CXL was most effective. Higher CXL efficiencies anteriorly are also typically observed using IVCM and AS-OCT and are related to the limited penetration depths of UVA in the tissue. Interestingly, increases in the AF intensity and lifetime were observed over time for the same corneal depth (Fig. 6). These alterations may also shed some light into the mechanism by which CXL induces corneal stiffening.

In conclusion, we have demonstrated that corneal stroma changes induced by CXL can be observed, in *ex vivo* tissue, as soon as 2 h after treatment based on the stroma AF intensity and lifetime. TPI imaging can provide a non-invasive, label-free, and fast evaluation of CXL outcome sooner than conventional clinical devices. Currently, the main limitations of TPI imaging *in vivo* are the need of contact interface, the high price, and the need for clinical certification in ophthalmology. Nevertheless, we hope to overcome these limitations and pave the way to introduce new devices based on TPI for clinical imaging of the human cornea. Such devices could highly improve ophthalmologic imaging, including an earlier assessment of expected CXL efficiency.

## References

[1] Hovakimyan M, Guthoff RF, Stachs O (2012). Collagen cross-linking: current status and future directions. J. Ophthalmol..

[2] Zhang X (2015). A review of collagen cross-linking in cornea and sclera. J. Ophthalmol..

[3] Zyablitskaya M (2017). Evaluation of therapeutic tissue crosslinking (TXL) for myopia using second harmonic generation signal microscopy in rabbit sclera. Investig. Opthalmology Vis. Sci..

[4] Khan YA (2011). Riboflavin and ultraviolet light a therapy as an adjuvant treatment for medically refractive Acanthamoeba keratitis: report of 3 cases. Ophthalmology.

[5] Wollensak G, Spoerl E, Seiler T (2003). Riboflavin/ultraviolet-a–induced collagen crosslinking for the treatment of keratoconus. Am J Ophthalmol.

[6] Raiskup-Wolf F, Hoyer A, Spoerl E, Pillunat LE (2008). Collagen crosslinking with riboflavin and ultraviolet-A light in keratoconus: long-term results. J Cataract Refract Surg.

[7] Jankov Ii MR, Jovanovic V, Delevic S, Coskunseven E (2011). Corneal collagen cross-linking outcomes: review. Open Ophthalmol. J..

[8] Caporossi A (2012). Riboflavin-UVA-induced corneal collagen cross-linking in pediatric patients. Cornea.

[9] Waszczykowska A, Jurowski P (2015). Two-year accelerated corneal cross-linking outcome in patients with progressive keratoconus. Biomed Res. Int..

[10] Lagali, N. *et al*. Laser-scanning *in vivo* confocal microscopy of the cornea: imaging and analysis methods for preclinical and clinical applications. In *Confocal Laser Microscopy - Principles and Applications in Medicine, Biology*, and *the Food Sciences* (ed. Lagali, N.), 10.5772/50821, (InTech, 2013).

[11] Koller T, Mrochen M, Seiler T (2009). Complication and failure rates after corneal crosslinking. J. Cataract Refract. Surg..

[12] Baenninger PB, Bachmann LM, Wienecke L, Kaufmann C, Thiel MA (2014). Effects and adverse events after CXL for keratoconus are independent of age: a 1-year follow-up study. Eye.

[13] Antoun J (2015). Rate of corneal collagen crosslinking redo in private practice: risk factors and safety. J. Ophthalmol..

[14] Mazzotta C (2013). Qualitative investigation of corneal changes after accelerated corneal collagen cross-linking (A-CXL) by *in vivo* confocal microscopy and corneal OCT. J. Clin. Exp. Ophthalmol..

[15] Mazzotta C, Traversi C, Caragiuli S, Rechichi M (2014). Pulsed vs continuous light accelerated corneal collagen crosslinking: *in vivo* qualitative investigation by confocal microscopy and corneal OCT. Eye.

[16] Zare M (2016). Effects of corneal collagen crosslinking on confocal microscopic findings and tear indices in patients with progressive keratoconus. Int. J. Prev. Med..

[17] Ozgurhan EB (2014). Evaluation of corneal stromal demarcation line after two different protocols of accelerated corneal collagen cross-linking procedures using anterior segment optical coherence tomography and confocal microscopy. J. Ophthalmol..

[18] Yam JCS, Chan CWN, Cheng ACK (2012). Corneal collagen cross-linking demarcation line depth assessed by visante OCT after CXL for keratoconus and corneal ectasia. J. Refract. Surg..

[19] Malta JBN, Renesto AC, Moscovici BK, Soong HK, Campos M (2015). Stromal demarcation line induced by corneal cross-linking in eyes with keratoconus and nonkeratoconic asymmetric topography. Cornea.

[20] Batista A, Breunig HG, Uchugonova A, Morgado AM, König K (2016). Two-photon spectral fluorescence lifetime and second-harmonic generation imaging of the porcine cornea with a 12 femtosecond laser microscope. J. Biomed. Opt..

[21] Batista Ana, Breunig Hans Georg, Donitzky Christoph, König Karsten, König Karsten (2018). 16 Two-photon microscopy and fluorescence lifetime imaging of the cornea. Multiphoton Microscopy and Fluorescence Lifetime Imaging.

[22] König K, Batista A, Hager T, Seitz B (2017). Multiphoton tomography of the human eye. Proc. SPIE.

[23] Batista A (2018). Assessment of human corneas prior to transplantation using high-resolution two-photon imaging. Investig. Opthalmology Vis. Sci..

[24] Batista A (2018). High-resolution, label-free two-photon imaging of diseased human corneas. J. Biomed. Opt..

[25] Batista, A., Breunig, H. G., Hager, T., Seitz, B. & König, K. Follow-up of accelerated-crosslinking non-invasively and label-free using multiphoton tomography. In *Proc. SPIE* 108581X–1:7, 10.1117/12.2509359 (2019).

[26] Georgakoudi I, Quinn KP (2012). Optical imaging using endogenous contrast to assess metabolic state. Annu Rev Biomed Eng.

[27] Quinn KP (2013). Quantitative metabolic imaging using endogenous fluorescence to detect stem cell differentiation. Sci. Rep..

[28] Batista, A., Breunig, H. G., König, A., Morgado, A. M. & König, K. Assessment of the metabolism and morphology of the porcine cornea, lens and retina by 2-photon imaging. *J. Biophotonics* **11**, e201700324:1–8 (2018). 10.1002/jbio.201700324 29575612

[29] Scarcelli G, Pineda R, Yun SH (2012). Brillouin optical microscopy for corneal biomechanics. Investig. Ophthalmol. Vis. Sci..

[30] Scarcelli G (2013). Brillouin microscopy of collagen crosslinking: Noncontact depth-dependent analysis of corneal elastic modulus. Investig. Ophthalmol. Vis. Sci..

[31] Kwok SJJ (2016). Selective two-photon collagen crosslinking *in situ* measured by Brillouin microscopy. Optica.

[32] Webb JN, Su JP, Scarcelli G (2017). Mechanical outcome of accelerated corneal crosslinking evaluated by Brillouin microscopy. J. Cataract Refract. Surg..

[33] Bueno JM (2011). Multiphoton microscopy of *ex vivo* corneas after collagen cross-linking. Invest Ophthalmol Vis Sci.

[34] Tan HY (2013). Characterizing the morphologic changes in collagen crosslinked-treated corneas by Fourier transform-second harmonic generation imaging. J Cataract Refract Surg.

[35] McQuaid R, Li J, Cummings A, Mrochen M, Vohnsen B (2014). Second-harmonic reflection imaging of normal and accelerated corneal crosslinking using porcine corneas and the role of intraocular pressure. Cornea.

[36] Mercatelli, R. *et al*. Characterization of the lamellar rearrangement induced by cross-linking treatment in keratoconic corneal samples imaged by SHG microscopy. In *Proc. SPIE* **10045**, 100450L:1–6 (2017).

[37] Mercatelli R (2016). Three-dimensional mapping of the orientation of collagen corneal lamellae in healthy and keratoconic human corneas using SHG microscopy. J. Biophotonics.

[38] Steven P, Hovakimyan M, Guthoff RF, Hüttmann G, Stachs O (2010). Imaging corneal crosslinking by autofluorescence 2-photon microscopy, second harmonic generation, and fluorescence lifetime measurements. J Cataract Refract Surg.

[39] Berezin MY, Achilefu S (2010). Fluorescence Lifetime Measurements and Biological Imaging. Chem. Rev. (Washington, DC, U. S.).

[40] König K (2008). Clinical multiphoton tomography. J. Biophotonics.

[41] Manickavasagam A (2014). Multimodal optical characterisation of collagen photodegradation by femtosecond infrared laser ablation. Analyst.

[42] Lutz V (2012). Impact of collagen crosslinking on the second harmonic generation signal and the fluorescence lifetime of collagen autofluorescence. Ski. Res. Technol..

